# Characterization of the complete chloroplast genome of the endangered and endemic bornean fruit *Artocarpus tamaran* Becc

**DOI:** 10.3389/fpls.2024.1513364

**Published:** 2024-12-12

**Authors:** Reni Lestari, Mahat Magandhi, Muhammad Rifqi Hariri, Ikhsan Noviady, Aditya Nugroho, Fitri Indriani

**Affiliations:** ^1^ Research Center for Applied Botany, National Research and Innovation Agency, Bogor, Indonesia; ^2^ Research Center for Biosystematics and Evolution, National Research and Innovation Agency, Bogor, Indonesia

**Keywords:** conservation, illumina, Moraceae, plastid genome, underutilized fruit

## 1 Introduction

*Artocarpus tamaran* Becc. is a member of the *Artocarpus* genus of the Moraceae family, comprising 74 plant species (POWO, 2024). The species tree may attain a height of 45 m and a stem diameter of 1 m, with buttresses up to 3 m in height (Kochummen, 2000). The species is endemic to Borneo, occurring in Sarawak, Sabah, Kalimantan, and Brunei Darussalam, specifically in low land to the hilly mixed Dipterocarpaceae forest, beside the river, on sandstone, clay, and alluvial substrate (POWO, 2024; Jarrett, 1959). It has also been recorded in the primary or old secondary forests and logged forests at 20 m to 1800 m above sea level (Jarrett, 1959). According to the Red List category of the International United Conservation Nations (IUCN), *Artocarpus tamaran* is classified as Vulnerable A2c according to the Red List category of the International Union for Conservation of Nature (IUCN, 2024). The species is endangered due to habitat loss, which has been converted into plantations, logged, burnt down, and climate affected such as in Sabah, Sarawak, and Kalimantan (IUCN, 2024; POWO, 2024). The species is utilized for fiber material sourced from the bark, which is used to produce cloth and hats (Kulip, 2003; Fern 2014), fresh fruit, and edible seed after being boiled or roasted (Lim, 2012). The stem, referred to as “terap” in local terminology, has potential applications in construction (Kochummen, 2000). The log and timber prices of the species were 22.90 USD m^-3^ and 50.88 USD m^-3^, respectively (Karmini et al., 2020).

The chloroplast genome displays a quadripartite structure and is circular. The structure comprises a large single-copy region (LSC) and a small single-copy region (SSC), separated by a pair of inverted repeats (IRs), with some exceptions noted where the loss of an IR or the SSC has occurred. The size of the chloroplast genome in terrestrial plants ranges from 19 to 217 kb, with the IRs generally measuring 20-26 kb in length (http://www.ncbi.nlm.nih.gov/genome/organelle). The chloroplasts proteome consists of around 3000 proteins that play roles in photosynthesis, and the biosynthesis of fatty acids, amino acids, hormones, vitamins, nucleotides, and secondary metabolites (Dobrogojski et al., 2020). The advancement and utilization of chloroplast genome engineering technology may inform the investigation of chloroplast gene functions, gene editing, gene expression regulation, and genome analysis (An et al., 2022). Regulation of chloroplast gene expression in chloroplast genome engineering is employed to achieve high-value industrial targets, improve photosynthetic capacity, and biofortify food crops (Boynton et al., 1988). This study presents the results of the chloroplast genome sequencing of the *A. tamaran* species.

## 2 Method

### 2.1 Plant material, DNA extraction and sequencing

A sample of *A. tamaran* was obtained from the living collection of Bogor Botanical Gardens in West Java, designated with collector number IN577. The plant sample originated from Central Kalimantan. Genomic DNA was extracted from fresh leaves utilizing the CTAB (cetyltrimethylammonium bromide) method as described by Doyle and Doyle (1987). The initial quantification and purity of DNA were evaluated using a Nanodrop 2000 (Thermo Scientific) and visualized through agarose gel electrophoresis with 1% TBE agarose. The Qubit dsDNA HS Assay Kit (Thermo Scientific) was utilized for enhanced DNA quantification accuracy. The integrity of DNA was assessed utilizing the 4150 TapeStation (Agilent).

Genomic DNA was utilized as the input for library preparation. The genomic DNA was enzymatically fragmented to obtain the required insert size. The fragmented DNA was ligated with MGI-compatible adapters, each containing a unique barcode for each sample. PCR was performed to amplify the library. The quality and quantity of library samples were assessed using Tape Station and Qubit Fluorometer, respectively. The amplified library samples underwent circularization, and the resulting circular DNA served as input for the DNB formation process. The DNBs were loaded onto the flow cell, and sequencing was conducted for 612 cycles (PE300) utilizing the MGI DNBSEQ-G400.

### 2.2 Chloroplast genome assembly and annotation

Quality control was conducted to evaluate the quality of reads utilizing FASTQC software version 0.11.8 (Andrews, 2010). Low-quality bases (less than 30), adapters, nucleotide position biases at the 3’ and 5’ ends, and sequence contamination were eliminated through trimming and filtering with Trimmomatic version 0.39. The parameters used were TruSeq3-PE.fa:2:30:10, SLIDINGWINDOW:4:28, LEADING:28, TRAILING:28, and MINLEN:20 (Bolger et al., 2014). The clean reads were then assembled using GetOrganelle version 1.7.7.1 (Jin et al., 2020). Annotation was conducted with CPGAVAS2 (http://47.96.249.172:16019/analyzer/annotate) (Shi et al., 2019), utilizing the cp genome of *Artocarpus gomezianus* Wall. ex Trécul (accession number: NC_080592) as a reference. This was followed by manual verification in Unipro Ugene v. 45.1 (Okonechnikov et al., 2012) and NCBI Genomic Workbench v. 3.8.2 (Kuznetsov and Bollin, 2021). The circular genome was visualized with OrganellarGenomeDRAW (OGDRAW) via the MPI-MP Chlorobox (Greiner et al., 2019).

## 3 Results

**Figure 1:**
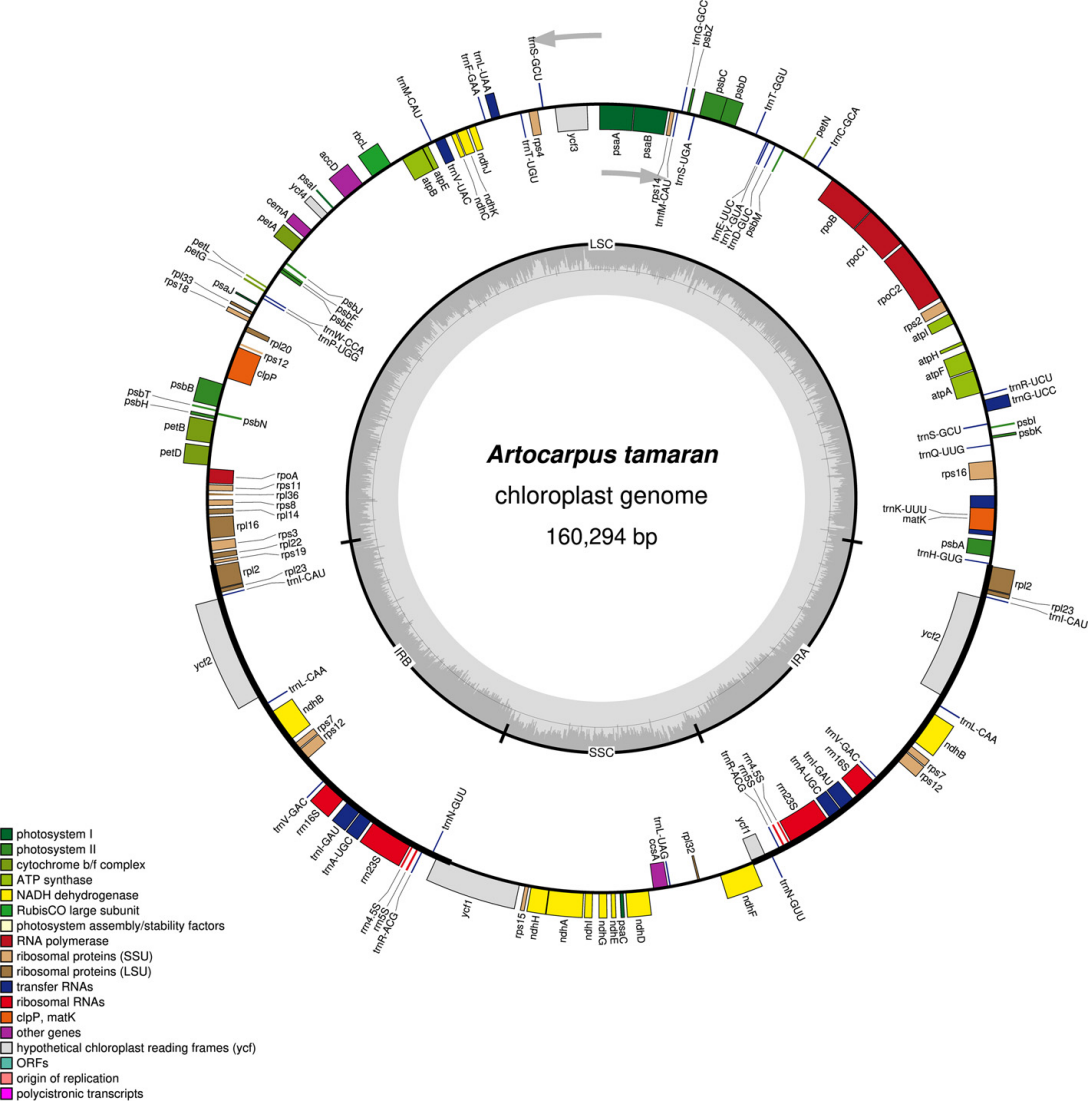
Gene map of the *A. tamaran* chloroplast genome.

The complete chloroplast genome of *A. tamaran* has been successfully assembled, measuring 160,294 bp and exhibiting a quadripartite structure comprising four regions: the large single-copy (LSC) region, the small single-copy (SSC) region, and two inverted repeats (IR) regions (**Figure 1**). The LSC region has a length of 88,789 bp, the SSC region measures 20,015 bp, and each IR region is 25,745 bp. The genome exhibits a total GC content of 36%, with the highest concentration observed in the IR regions at 46.2%, followed by the LSC region at 34.2% and the SSC region at 28.9%. A total of 129 genes, comprising 110 unique genes, were annotated in the *A. tamaran* chloroplast genome. The identified genes comprised 84 protein-coding genes (77 unique), 37 tRNAs (29 unique), and 8 rRNAs (4 unique). Of the 129 genes analyzed, 14 exhibited a single intron, while three genes (*rps*12, *ycf*3 and *clp*P) contained two introns (see **Table 1**).

**Table 1:** List of genes in the *A. tamaran* chloroplast genome.

Functional category	Group of Gene	Name of Gene
Self-replication	rRNA	*rrn*16^d^, *rrn*23^d^, *rrn*4.5^d^, *rrn*5^d^
	tRNA	*trn*K-UUU*, *trn*Q-UUG, *trn*S-GCU^d^, *trn*R-UCU, *trn*C-GCA, *trn*D-GUC, *trn*Y-GUA, *trn*E-UUC, *trn*T-GGU, *trn*S-UGA, *trn*G-GCC, *trn*M-CAU, *trn*T-UGU, *trn*L-UAA*, *trn*F-GAA, *trn*fM-CAU, *trn*W-CCA, *trn*G-UCC, *trn*P-UGG, *trn*H-GUG, *trn*L-CAA^d^, *trn*V-GAC^d^, *trn*V-UAC*, *trn*I-GAU^d^*, *trn*A-UGC^d^*, *trn*R-ACG^d^, *trn*N-GUU^d^, *trn*L-UAG, *trn*I-CAU^d^
	Large subunit ribosomal proteins (LSU)	*rpl*14, *rpl*16*, *rpl*2^d^*, *rpl*20, *rpl*22, *rpl*23^d^, *rpl*32, *rpl*33, *rpl*36
	Small subunit ribosomal proteins (SSU)	*rps*11, *rps*12^d^**, *rps*14, *rps*15, *rps*16*, *rps*18, *rps*19, *rps*2, *rps*3, *rps*4, *rps*7^d^, *rps*8
	DNA-dependent RNA polymerase	*rpo*A, *rpo*B, *rpo*C1*, *rpo*C2
	Subunits of ATP synthase	*atp*A, *atp*B, *atp*E, *atp*F*, *atp*H, *atp*I
	Subunits of NADH-dehydrogenase	*ndh*A*, *ndh*B^d^*, *ndh*C, *ndh*D, *ndh*E, *ndh*F, *ndh*G, *ndh*H, *ndh*I, *ndh*J, *ndh*K
Photosynthesis	Subunits of photosystem I	*psa*A, *psa*B, *psa*C, *psa*I, *psa*J
	Subunits of photosystem II	*psb*A, *psb*B, *psb*C, *psb*D, *psb*E, *psb*F, *psb*H, *psb*I, *psb*J, *psb*K, *psb*M, *psb*N, *psb*T, *psb*Z, *ycf*3**
	Subunits of cytochrome b/f complex	*pet*A, *pet*B*, *pet*D*, *pet*G, *pet*L, *pet*N
	Subunit rubisco	*rbc*L
	Subunit of acetyl-CoA-carboxylase	*acc*D
	C-type cytochrome synthesis gene	*ccs*A
Other function	Protease	*clp*P**
	Maturase	*mat*K
	Envelope membrane protein	*cem*A
Unknown function	Conserved open reading frames	*ycf*1^d^, *ycf*2^d^, *ycf*4

^d^, gene duplication; *, single intron; **, double intron.

## Data Availability

This study analyzes datasets available in the NCBI Short Read Archive (SRA) under accession number SRR31020103 (https://www.ncbi.nlm.nih.gov/sra/SRR31020103). The BioProject and Bio-Sample numbers are PRJNA1173771 and SAMN44319506, respectively. The chloroplast genome sequence of *A. tamaran* has been deposited in the NCBI under accession number PQ493654.
